# Mutations in the riboflavin biosynthesis pathway confer resistance to furazolidone and abolish the synergistic interaction between furazolidone and vancomycin in Escherichia coli

**DOI:** 10.1099/mgen.0.001356

**Published:** 2025-02-11

**Authors:** Hannah Wykes, Vuong Van Hung Le, Jasna Rakonjac

**Affiliations:** 1 School of Food Technology and Natural Sciences, Massey University, Palmerston North, New Zealand; 2 Living Systems Institute, University of Exeter, Exeter, UK; 3 Faculty of Health and Life Sciences, University of Exeter, Exeter, UK

**Keywords:** antibiotic resistance, furazolidone, riboflavin, synergy, vancomycin

## Abstract

The combined application of furazolidone and vancomycin has previously been shown to be synergistic against Gram-negative pathogens, with great therapeutic promise. However, the emergence and mechanism of resistance to this antibiotic combination have not been characterized. To fill this gap, we here selected *Escherichia coli* progeny for growth on the furazolidone–vancomycin combination at the concentration where the parent was sensitive. We show that selected clones were associated with increased resistance to neither, only one drug, or both furazolidone and vancomycin, but in all cases were associated with a decrease in the growth inhibition synergy. Using whole-genome sequencing, we identified various gene mutations in the resistant mutants. We further investigated the mechanism behind the most frequently arising mutations, those in the riboflavin biosynthesis genes *ribB* and *ribE*, that represent novel mutations causing furazolidone resistance and diminished vancomycin–furazolidone synergy. It was found that these *ribB/ribE* mutations act predominantly by decreasing the activity of the NfsA and NfsB nitroreductases. The emergence of the *ribB*/*ribE* mutations imposes a significant fitness cost on bacterial growth. Surprisingly, supplementing the medium with riboflavin, which compensates for the affected riboflavin biosynthesis pathway, could restore the normal growth of the *ribB*/*ribE* mutants while having no effects on the furazolidone resistance phenotype. Searching the *ribB/ribE* mutations in the public sequencing database detects the presence of the furazolidone-resistance-conferring *ribE* mutations (TKAG^131–134^ deletion or duplication) in clinical isolates from different countries. Hypotheses explaining why these *ribE* mutations were found in clinical isolates despite having poor fitness were further discussed.

**Impact Statement**

Antibiotic resistance is a serious threat to human health. With a drying antibiotic development pipeline to counteract antibiotic resistance, synergistic combinations of approved antibiotics provide a promising approach to deliver efficient antibacterial therapy in a timely manner. In the present work, we characterized the resistance emergence and mechanisms towards the synergistic combination of two approved antibiotics, furazolidone and vancomycin, in *Escherichia coli*. Understanding the types of genetic mutations that can emerge and how they affect drug interaction would inform the drug dosing strategy to treat bacterial infections for a desirable outcome. In addition, we described novel mutations in the riboflavin biosynthesis pathway that confer resistance to furazolidone, a nitrofuran antibiotic, and should be included in future nitrofuran resistance epidemiology studies besides well-known targets such as *nfsA*, *nfsB* and *ribE*.

## Data Summary

Sequencing data analysed in this work are available in the National Center for Biotechnology Information (NCBI) database under the BioProject PRJNA854676. (The parental strain BW25113 is called K2653 in the sequencing data.) The authors confirm that all supporting data and protocols have been provided within the article.

## Introduction

Ever-increasing antibiotic resistance is a current and future global health issue, with the most urgent need identified by the World Health Organization to develop treatments for Gram-negative bacteria. Among multiple strategies being developed, synergistic antibiotic combinations are clinically important for several reasons. First, they lower the minimal effective dosage of each constituting drug, reducing side effects and toxicity while broadening available drug options by including drugs that would otherwise be toxic at the effective dose in a mono-therapy [1]. Second, synergistic combinations may be sufficient to kill mutants resistant to individual agents, suppressing the emergence of resistant mutants during combinatorial therapy [2]. Nonetheless, the latter would be less significant if mutations arise that confer cross-resistance to both antibacterials and abolish the synergistic interaction. It is therefore important, following the discovery of a synergistic pair, to evaluate the emergence and phenotypes of the resistant mutants. Notably, isolating, identifying and characterizing mutations that cause synergy loss may reveal the molecular mechanism behind the interaction [3,4].

We have previously reported that the combination of furazolidone, a nitrofuran antibiotic, and vancomycin displays antibacterial synergy in *Escherichia coli* [5]. This combination holds promise to repurpose vancomycin, a high-molecular-weight glycopeptide antibiotic that poorly translocates across the outer membrane and is prescribed for the treatment of Gram-positive infections, into a treatment option for infections with multidrug-resistant Gram-negative bacteria. Another potential application of this is during treatment for Gram-positive infections, such as *Clostridiodes difficile*; rather than treatment with only vancomycin, a vancomycin–furazolidone combination could be used to prevent *E. coli* outgrowth. With the same rationale, this combination can be prescribed for gastrointestinal *E. coli* infections while suppressing the potential emergence and outgrowth of *C. difficile*, a serious concern associated with oral antibiotic administration. In the present work, we isolated and characterized *E. coli* strains carrying mutations conferring resistance to the synergistic furazolidone–vancomycin combination, showing that the most frequent resistance mechanism was through the biosynthesis pathway of riboflavin, the precursor to the cofactors required for nitroreductases, enzymes responsible for furazolidone (prodrug) activation.

## Methods

### Growth conditions and antibiotics

*E. coli* strains were grown at 37 °C with shaking at 200 rotations per minute. Growth media were either cation-adjusted Mueller Hinton (CAMH), 2×YT (BD Difco™) or BD BBL™. They were used either as a liquid broth, or solid plates where agar (Pure Science) was added to a final concentration of 1% w/v to solidify the media. Stocks of antibiotics (GoldBio) were made in water (ampicillin, kanamycin and vancomycin) or DMSO (chloramphenicol and furazolidone).

### Bacterial strain construction

Bacterial strains and plasmids used in this study are shown in Table 1. Δ*nfsA* Δ*nfsB* double knockouts of isolated mutants were constructed by stepwise rounds of P1 bacteriophage transduction [6] using single-gene knockout mutations from the Keio collection as donors [7] followed by excision of the kanamycin resistance marker using flippase (FLP) recombination, as previously described [8]. *E. coli* strains transformed with pCA24N and derived plasmids from the ASKA collection [9] were grown in media containing 30 mg l^−1^ chloramphenicol, and expression was induced with 0.1 mM IPTG, unless otherwise specified. Riboflavin was supplemented in the media at a final concentration of 1 mM.

**Table 1.** Bacterial strains and plasmids used in this study

*E. coli* K12 laboratory strain and plasmid	Genotype/description	Source
**Strain**		
BW25113 (PS)∗	*rrnB3* Δ*lacZ4787 hsdR514* Δ(*araBAD)567* Δ(*rhaBAD)568 rph-1*	[43]
K2654	BW25113, no mutations identified	This study
K2655	BW25113, *nlpI* nonsense mutation	This study
K2657	BW25113, *opgG* frameshift	This study
K2658	BW25113, *rpoC* missense	This study
K2659	BW25113, *ftsH* frameshift	This study
K2660	BW25113, *ftsH* missense, *waaR*::IS*5*	This study
K2661	BW25113, *wecC* frameshift	This study
K2662 (B1)†	BW25113, *ribB* 5′ UTR point mutation	This study
K2663 (E1)†	BW25113, *ribE* insertion	This study
K2664	BW25113, *ftsH* frameshift	This study
K2665 (E2)†	BW25113, *ribE* deletion, *fabH* missense	This study
K2666 (E3)†	BW25113, *ribE* deletion, *yjhQ* missense	This study
K2667 (E4)†	BW25113, *ribE* deletion	This study
K2668 (E5)†	BW25113, *ribE* deletion, *ycjM* missense	This study
K2669 (B2)†	BW25113, *ribB* promoter::IS*1*	This study
K2670 (B3)†	BW25113, *ribB* promoter::IS*5*	This study
K2671 (B4)†	BW25113, *ribB* 5′ UTR point mutation	This study
K2642 (PS+*ribE*)	BW25113 (pCA24N::*ribE*)	This study
K2643 (E1+*ribE*)	K2663 (pCA24N::*ribE*)	This study
K2644 (E2+*ribE*)	K2665 (pCA24N::*ribE*)	This study
K2645 (E3+*ribE*)	K2666 (pCA24N::*ribE*)	This study
K2646 (E4+*ribE*)	K2667 (pCA24N::*ribE*)	This study
K2647 (E5+*ribE*)	K2668 (pCA24N::*ribE*)	This study
K2648 (PS+*ribB*)	BW25113 (pCA24N::*ribB*)	This study
K2649 (B1+*ribB*)	K2662 (pCA24N::*ribB*)	This study
K2650 (B2+*ribB*)	K2669 (pCA24N::*ribB*)	This study
K2651 (B3+*ribB*)	K2670 (pCA24N::*ribB*)	This study
K2652 (B4+*ribB*)	K2671 (pCA24N::*ribB*)	This study
K2709 (PS knockout)	BW25113 Δ*nfsA* Δ*nfsB*	This study
K2710 (E1 knockout)	K2663 Δ*nfsA* Δ*nfsB*	This study
K2711 (E4 knockout)	K2667 Δ*nfsA* Δ*nfsB*	This study
K2712 (B2 knockout)	K2669 Δ*nfsA* Δ*nfsB*	This study
K2713 (B3 knockout)	K2670 Δ*nfsA* Δ*nfsB*	This study
**Plasmid**		
pCP20	AMP^r^, CHL^r^, FLP^+^, λ cI857^+^, λ *p*_R_ Rep^ts^ (for mediating FLP recombination)	[44]
pCA24N::*ribB*	CHL^r^; *lacI*^q^, pCA24N P_T5-lac_::*ribB*	[9]
pCA24N::*ribE*	CHL^r^; *lacI*^q^, pCA24N P_T5-lac_::*ribE*	[9]

* The BW25113 strain used for the selection in this study had gained a spontaneous IS*1* insertion mutation in the *gtrB* gene in comparison to the reference sequence (GenBank accession no. CP009273.1) and will be referred to as parental strain (PS). The IS*1* mutation was also present in all selected strains.

† The *ribB* and *ribE* mutants will be referred to as B1-B4 and E1-E5

### Antimicrobial susceptibility assays and growth rate assays

Antibiotic minimum inhibitory concentrations (MICs) were determined according to the Clinical and Laboratory Standards Institute guidelines [10] using broth microdilution and agar dilution methods. Growth rate assays were conducted for the broth microdilution assays, with the optical density at 600 nm (OD_600_) measured every hour for either 24 or 48 h (Multiskan™ GO Microplate Spectrophotometer).

### Growth inhibition checkerboard assays

Checkerboard assays were used to assess how the furazolidone–vancomycin interaction inhibits *E. coli* growth by standard microdilution method. Assays were conducted in CAMH broth in 384-well microplates. Twofold serial dilutions of furazolidone and vancomycin were used. Each well contained 5×10^5^ c.f.u. ml^−1^, 1% DMSO and antibiotics in a final volume of 50 µl. The microplates were incubated at 37 °C, and the OD_600_ was measured after 18 h (Multiskan™ GO Microplate Spectrophotometer). Each treatment was performed in triplicate, and the lowest drug concentration that caused a mean growth inhibition of at least 90% compared to the no-antibiotic control was defined as the MIC [11].

Fractional inhibitory concentration index (FICI) was calculated as follows:


$$FICI=\frac{MIC_{FZ}\left(combination\right)}{MIC_{FZ}\left(alone\right)}+\frac{MIC_{VAN}\left(combination\right)}{MIC_{VAN}\left(alone\right)}$$

Where MIC_FZ_(combination) and MIC_VAN_(combination) are the MICs for furazolidone and vancomycin when used in combination, and MIC_FZ_(alone) and MIC_VAN_(alone) are the MICs for furazolidone and vancomycin when used alone. The lowest FICI values were used to determine interactions: FICI≤0.5 indicates synergy, FICI>4 indicates antagonism and 0.5<FICI≤4 indicates additivity [12].

### Isolating resistant mutants

Mutants of *E. coli* strain BW25113 were selected on CAMH agar containing a combination of vancomycin (256 mg l^−1^) and furazolidone (2 mg l^−1^). Twenty independent overnight cultures each inoculated from single colonies were separately spread on 20 selection plates. Briefly, 100 µl of each overnight culture was added to 2.5 ml of molten 0.5% CAMH agar (at ~47 °C), vortexed, and then poured onto the selective plate. Bacterial colonies were observed after 48 h incubation and then sub-streaked onto non-selective agar plates. To minimize the chance of isolating colonies with identical mutations, only one colony was picked from each plate unless differences in colony morphology were observed.

### Comparative genome analysis

Genomic DNA was extracted using the DNeasy UltraClean Microbial Kit (Qiagen, Cat. No. 10196–4) according to the manufacturer’s instructions. The samples were submitted for whole-genome sequencing to Massey Genome Service (Massey University, Palmerston North, New Zealand). Libraries were prepared using the Illumina DNA Prep kit and sequenced on the Illumina MiSeq 2×250 base-paired-end v2 platform.

The raw reads were trimmed to an error probability cut-off of 0.001 (Phred score of 30), and reads less than 25 bases were removed using SolexaQA++ v3.1.7.1 [13]. The trimmed reads were aligned to the reference genome (*E. coli* BW25113 accession no. CP009273.1) [14] using bowtie2 v2.4.2 [15] in the --very-sensitive mode. SAMtools v1.14 [16] was used to convert the SAM sequence alignment files into BAM files, followed by variant calling using freebayes v1.3.1 [17], with the ploidy set to 1. The variants were annotated using SnpEff v4.4.20(1) [18].

Genomic structural variations were identified by extracting the unmapped reads using SAMtools v1.14 [16], which were then assembled into contigs using SPAdes v3.13.0 [19], using the careful mode. The generated contigs were mapped to the reference *E. coli* BW25113 genome using the National Centre for Biotechnology Information (NCBI) nucleotide blast+ 2.120.0 [20] to determine the location of any structural variations, if present.

### RNA and protein modelling

The homology-based secondary structural model of the flavin mononucleotide (FMN) aptamer at the 5′-untranslated region of the *ribB* mRNA (corresponding to the reverse strand at the coordinates 3177808–3178077 of the reference *E. coli* BW25113 genome) was extracted from [21] and visualized using Varne v3.9 [22]. One of the 12 pentamers that form the 60-subunit RibE icosahedral biological complex was modelled using ColabFold v1.3.0 with default parameters [23] with the input being five copies of the RibE amino acid sequence (GenBank accession no. AIN30914.1) separated by colons.

### Nitroreductase activity assays

Nitroreductase activity assays [24] were conducted on cell extracts of selected *ribB*/*ribE* mutants, the PS and the corresponding *ribB*/*ribE* complemented strains. Each strain was analysed in three independent assays.

Overnight cultures were diluted 1 : 100 into 25 ml of CAMH broth and grown to OD_600_ ~0.5 at 37 °C, centrifuged (10 min, 4000 ***g***) and the pellets were stored at −20 °C until use. The pellets were washed with 10 ml of pre-chilled 50 mM Tris-HCl [tris(hydroxymethyl)aminomethane hydrochloride; pH 7.4], centrifuged (10 min, 4000 ***g***, 4 °C) and resuspended in 3.5 ml of pre-chilled 50 mM Tris-HCl (pH 7.4). The OD_600_ of each cell suspension was measured and adjusted with 50 mM Tris-HCl to a concentration of 1×10^9^ c.f.u. ml^−1^. Next, 3 ml of this cell suspension was sonicated (amplitude 15 for 4 min, 2 s on, 2 s off) using the microtip of a Virsonic 600 ultrasonic cell disruptor (Qsonica, part no. Q700). The cell lysate was then centrifuged (14 000 ***g***, 30 min, room temperature), and the supernatant was collected for enzymatic analyses.

Nitroreductase activity assays were performed on a 96-well plate, and each reaction was performed in triplicate. Each well contained 0.1 mM nicotinamide adenine dinucleotide phosphate (NADPH) (Roche), 0.1 mM furazolidone and 50 µl cell extract in 50 mM Tris-HCl (pH 7.4) in a total volume of 200 µl. NADPH was added last to initiate the reaction. Wells without cell extract were used as negative controls. The assay was incubated at 25 °C, and absorbance at 400 nm was measured every minute for 12 h.

### Searching for the *ribB/ribE* mutations in clinical isolates

For the TKAG deletion/duplication mutations found in the *ribE* mutants, the corresponding RibE amino acid sequence (GenBank accession no. AIN30914.1 with TKAG deletion/duplication) was queried against the NCBI non-redundant protein sequence using the blastp web server [20]. For the mutation in the 5′-untranslated region of *ribB*, the corresponding mutated nucleotide sequence ranging from 3177808 to 3178077 of the reference genome BW25113 was queried against the GenBank nucleotide collection using megaBlast with default parameters [25]. For the insertional mutation within the promoter of the *ribB* gene, an *in silico* PCR was used. A pair of primers targeting the *ribB* promoter was designed using the primerBlast web server [26], 5′-GGTTACCAGAATCAGGGCAGT-3′ and 5′- GTTGAGTGCCATTGTAGTGCG-3′, and then queried using the same tool with default parameters except setting *E. coli* as the searching database to predict the amplicon size. The amplicon size of the wild-type was predicted to be 324 bp, while the mutants containing IS*1*/IS*5* within the *ribB* promoter were expected to have a larger amplicon by 0.8–1.2 kb. Noteworthily, this *in silico* PCR would not detect the transpositional mutations for incomplete fragmented genome assemblies. If any *E. coli* isolate containing IS*1*/IS*5* transposition within the *ribB* promoter in the database was sequenced and assembled with a short-read sequencing technique only, the genome assembly would be fragmented at the insertional site due to the presence of multiple copies of the IS*1*/IS*5* elements in a genome; therefore, the *in silico* PCR would fail to generate a correct amplicon.

The bacterial genome assembly containing the queried mutation was retrieved and searched against the Comprehensive Antibiotic Resistance Database with default parameters to identify the presence of other antimicrobial resistance (AMR) genes [27].

## Results

### Selecting antibacterial resistance mutations to the synergistic furazolidone–vancomycin combination

To isolate mutants resistant to the furazolidone–vancomycin combination, stationary-phase overnight cultures of BW25113 PS were spread on selective agar plates containing a combination of 256 mg l^−1^ vancomycin and 2 mg l^−1^ furazolidone (Fig. S1, available in the online version of this article). Overall, 17 resistant mutants were isolated and sequenced (Table 2). Different mutation types were found, including nonsense (*nlpI*), missense (*rpoC*), frameshift (*ftsH*, *wecC*, *opgG*), in-frame deletion (*ribE*), IS*1*/IS*5* insertions and point mutations in the 5′ untranslated region of *ribB*. Notably, most of the isolated resistant mutants were shown to contain mutations in essential genes: *ribB* (×4), *ribE* (×5), *ftsH* (×3) and *rpoC* (×1).

**Table 2.** Mutations identified in isolated furazolidone–vancomycin-resistant mutants in reference to the BW25113 genome (accession no. CP009273.1)

Strain**∗**	Mutation	Gene	Predicted effect
K2654	No mutations identified†		
K2655	3302181 (G→A)	*nlpI*	NlpI (Lipoprotein NlpI) Gln35Stop
K2657	1105355 (A insertion)	*opgG*	OpgG (Glucans biosynthesis protein G) frameshift at codon 190
K2658	4178876 (A→G)	*rpoC*	RpoC (DNA-directed RNA polymerase subunit beta′) missense Glu1200Gly
K2659	Δ3319614–3319624	*ftsH*	FtsH (ATP-dependent zinc metalloprotease FtsH) frameshift at codon 224
K2660	3318752 (G→A) (+*waaR* IS*5* insertion)	*ftsH*	FtsH missense Pro515Ser
K2661	Δ3964950	*wecC*	WecC (UDP-*N*-acetyl-d-mannosamine dehydrogenase) frameshift at codon 112
K2662 (B1)	3177902 (C→A)	*ribB*	Single nucleotide substitution in *ribB* 5′ UTR (RibB: 3,4-dihydroxy-2-butanone 4-phosphate synthase)
K2663 (E1)	Duplication 430487–430498	*ribE*	RibE (6,7-dimethyl-8-ribityllumazine synthase) duplication of codons 131–134 (TKAG)
K2664	Δ3318882	*ftsH*	FtsH frameshift at codon 472
K2665 (E2)	Δ430487–430498 (+*fabH* missense Met65Ile)	*ribE*	RibE in-frame deletion of codons 131–134 (TKAG)
K2666 (E3)	Δ430487–430498 (+*yjhQ* missense Glu23Lys)	*ribE*	RibE in-frame deletion of codons 131–134 (TKAG)
K2667 (E4)	Δ430487–430498	*ribE*	RibE in-frame deletion of codons 131–134 (TKAG)
K2668 (E5)	Δ430487–430498 (+*ycjM* missense Gln57Lys)	*ribE*	RibE in-frame deletion of codons 131–134 (TKAG)
K2669 (B2)	3178086	*ribB*	IS*1* inserted in *ribB* promoter
K2670 (B3)	3178092	*ribB*	IS*5* inserted in *ribB* promoter
K2671 (B4)	3178074 (A→C)	*ribB*	Single nucleotide substitution in *ribB* 5′ UTR

* Sequencing showed that the parental strain (PS) unexpectedly had an IS*1* insertion in the *gtrB* gene at the start of the selection experiment. This *gtrB* mutation was also present in all derived resistant mutants.

† Chromosomal inversion or epigenetic changes in this strain have not been ruled out.

**Fig. 1.**
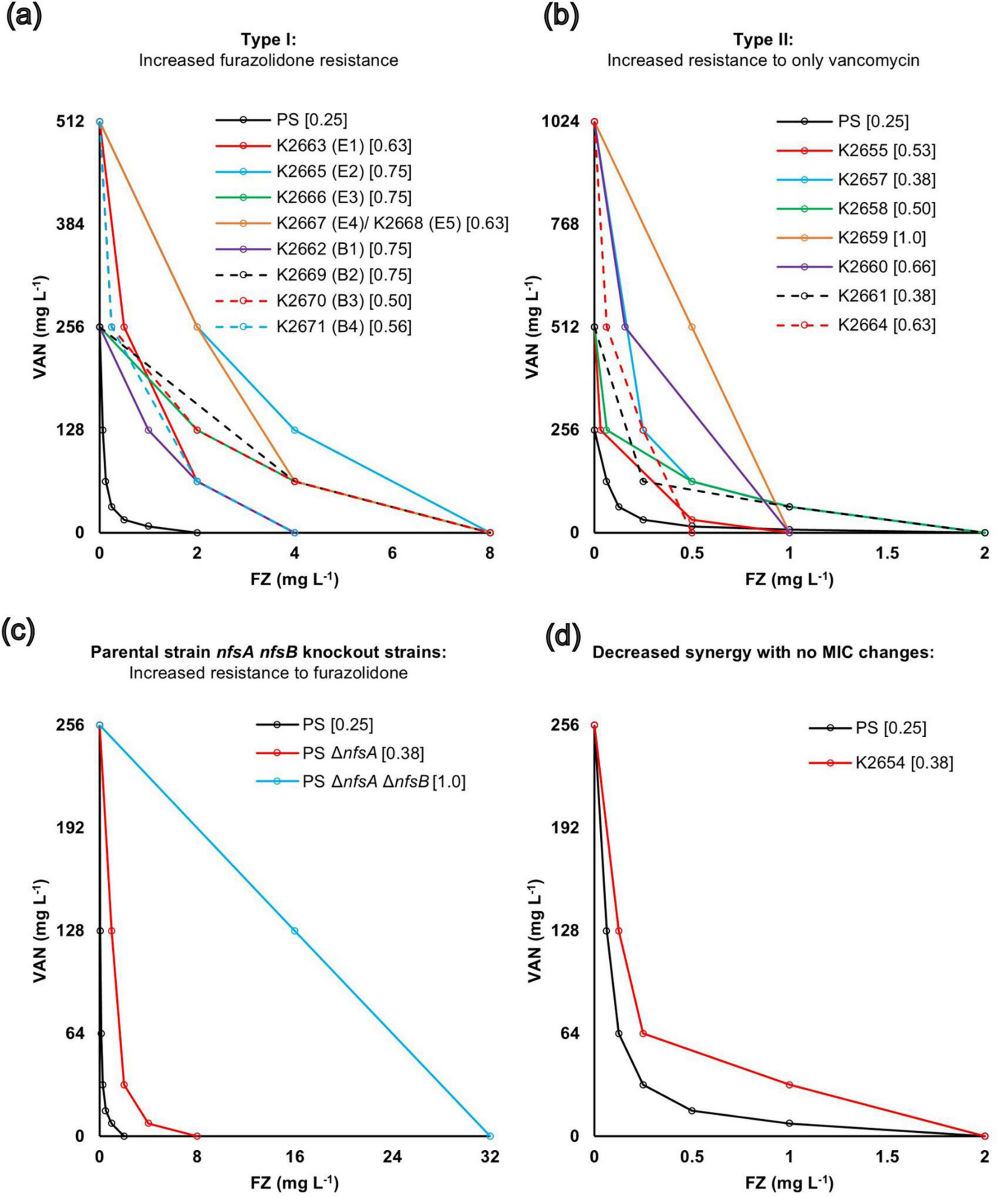
The vancomycin–furazolidone interaction in the isolated mutants. Checkerboard assays in liquid cultures were conducted on isolated resistant strains to construct the isobolograms for furazolidone–vancomycin interaction. All mutants had decreased synergy, reflected by their isobologram curve being less concave than the parental one. (**a**) Type I, increased furazolidone resistance (includes resistance to both furazolidone and vancomycin). (**b**) Type II, increased vancomycin resistance only. (**c**) The Δ*nfsA* and Δ*nfsA* Δ*nfsB* mutations in the PS, which are known to confer furazolidone resistance but were not found in our double-drug resistance selection experiment, are shown. (**d**) The isobologram for K2654, showed no MIC changes. Each point on the isobologram curve indicates the minimum concentration of each reagent in combination required to inhibit bacterial growth. The experiment was performed using three replicates, showing similar results. The FICI values for each strain are shown in square brackets. The *ribB* and *ribE* mutants are denoted by (B) and (E), respectively.

We also examined how individual antibiotic MICs and the furazolidone–vancomycin interaction changed in the isolated mutants, using antibiotic susceptibility broth microdilution and checkerboard assays, respectively. Strikingly, all isolated mutants demonstrated decreased synergy, whereas the changes in MICs for individual antibacterials fell into two main groups: (I) increased furazolidone resistance (with or without increased vancomycin resistance) and (II) increased vancomycin resistance only (Fig. 1). There was also one mutant that displayed decreased synergy with no individual MIC changes.

#### Mutations in the riboflavin biosynthesis pathway are associated with furazolidone resistance

**Fig. 2.**
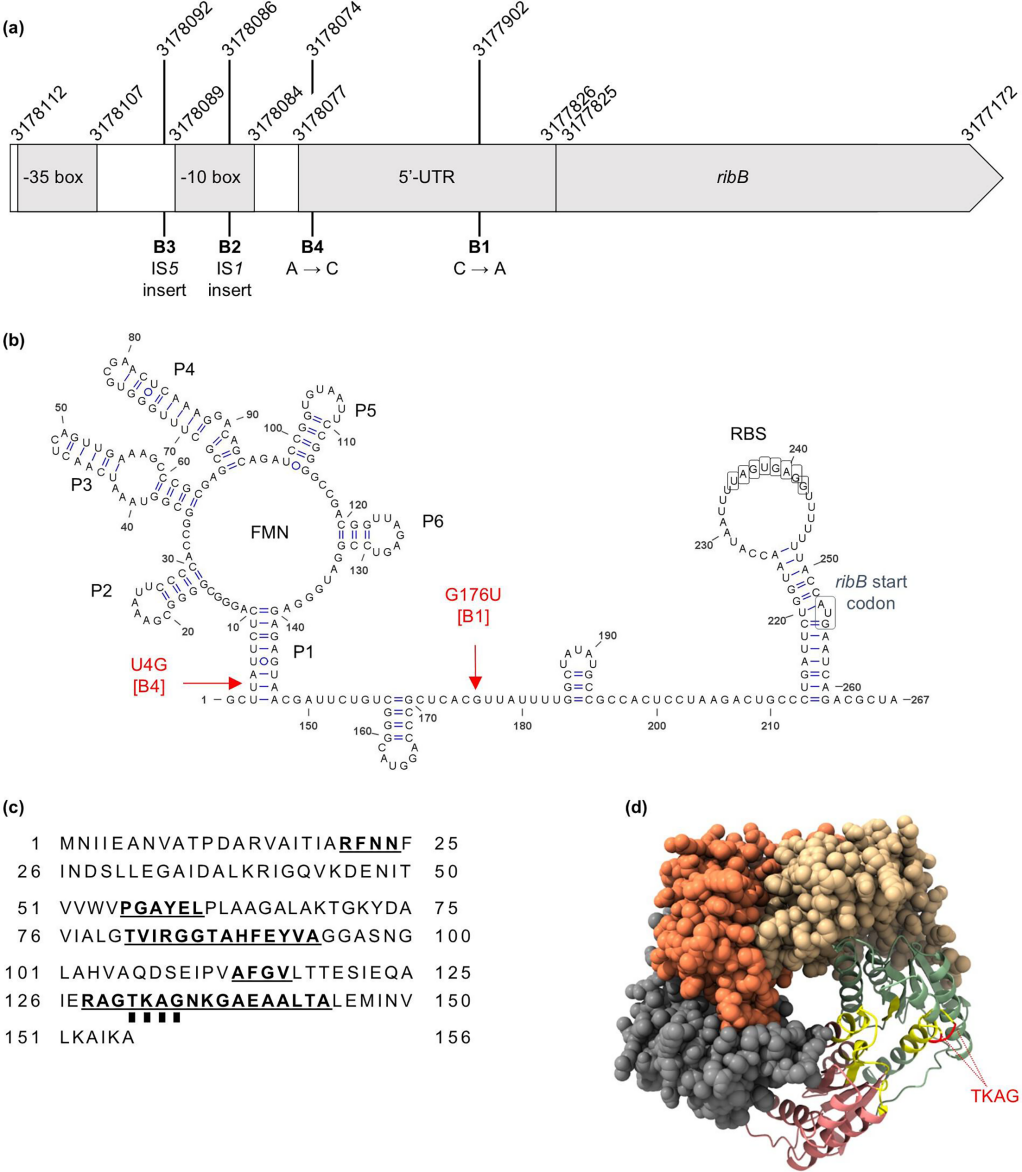
Annotation of the *ribB*/*ribE* mutations. (**a**) The position of the *ribB* mutations in strains B1, B2, B3 and B4. Mutants B1 and B4 had single nucleotide mutations in the mRNA 5′-UTR, which forms a regulatory riboswitch. Mutants B2 and B3 had IS*1* and IS*5* insertions, respectively, within the promotor region. The genome coordinates are used in accordance with the BW25113 reference genome (GenBank accession no. CP009273.1). Diagram not to scale. (**b**) Modelled secondary structure of the *ribB* riboswitch and the annotated mutations. (**c**) The amino acid sequence of the RibE protein. Residues making up the active site are emboldened and underlined [37]. The TKAG residues duplicated in strain E1, and deleted in strains E2, E3, E4 and E5, are marked by squares underneath the residue letters. (**d**) The ColabFold-predicted model of a RibE pentamer. Each RibE biological complex is an icosahedron composed of 60 monomeric units (=12 pentamers). The active site residues are coloured yellow, and the mutated TKAG stretch is coloured red. RBS, ribosome binding site.

Of the 17 isolated mutants, 9 contained *ribB* or *ribE* mutations encoding enzymes in the riboflavin biosynthesis pathway (Fig. S2). This pathway is responsible for the biosynthesis of FMN and flavin adenine dinucleotide (FAD), cofactors for the two major nitrofuran-activating nitroreductases, NfsA and NfsB, and minor nitroreductase AhpF [24,28, 29]. These mutants demonstrated up to a fourfold increase in MIC_FZ_ (Fig. 1). The *ribB* mutants all had mutations upstream of the coding region: B2 and B3 had IS*5* or IS*1* insertions in the promoter, and a fourfold MIC_FZ_ increase, while B1 and B4 had single nucleotide substitutions in the 5′ untranslated region (5′-UTR) of the *ribB* mRNA (Fig. 2a), and a twofold MIC_FZ_ increase. The 5′-UTR of the *ribB* gene is a highly structured regulatory riboswitch that, upon binding FMN, represses *ribB* expression at both the transcriptional and translational levels (Fig. 2b) [30].

Regarding the *ribE* mutants, the same four amino acids (TKAG) were either deleted (mutants E2, E3, E4 and E5) or duplicated (mutant E1) (Fig. 2c, Table 2), causing a fourfold or twofold increase in MIC_FZ_, respectively. We modelled a pentamer of the RibE icosahedron [23], showing that the TKAG residues are located at the interface of two adjacent monomeric subunits, in the active site of the complex (Fig. 2d). Duplication or deletion of the TKAG residues is therefore expected to negatively affect the RibE enzyme activity.

### Growth rates and furazolidone dose-response curves of the *ribB/ribE* mutants

**Fig. 3.**
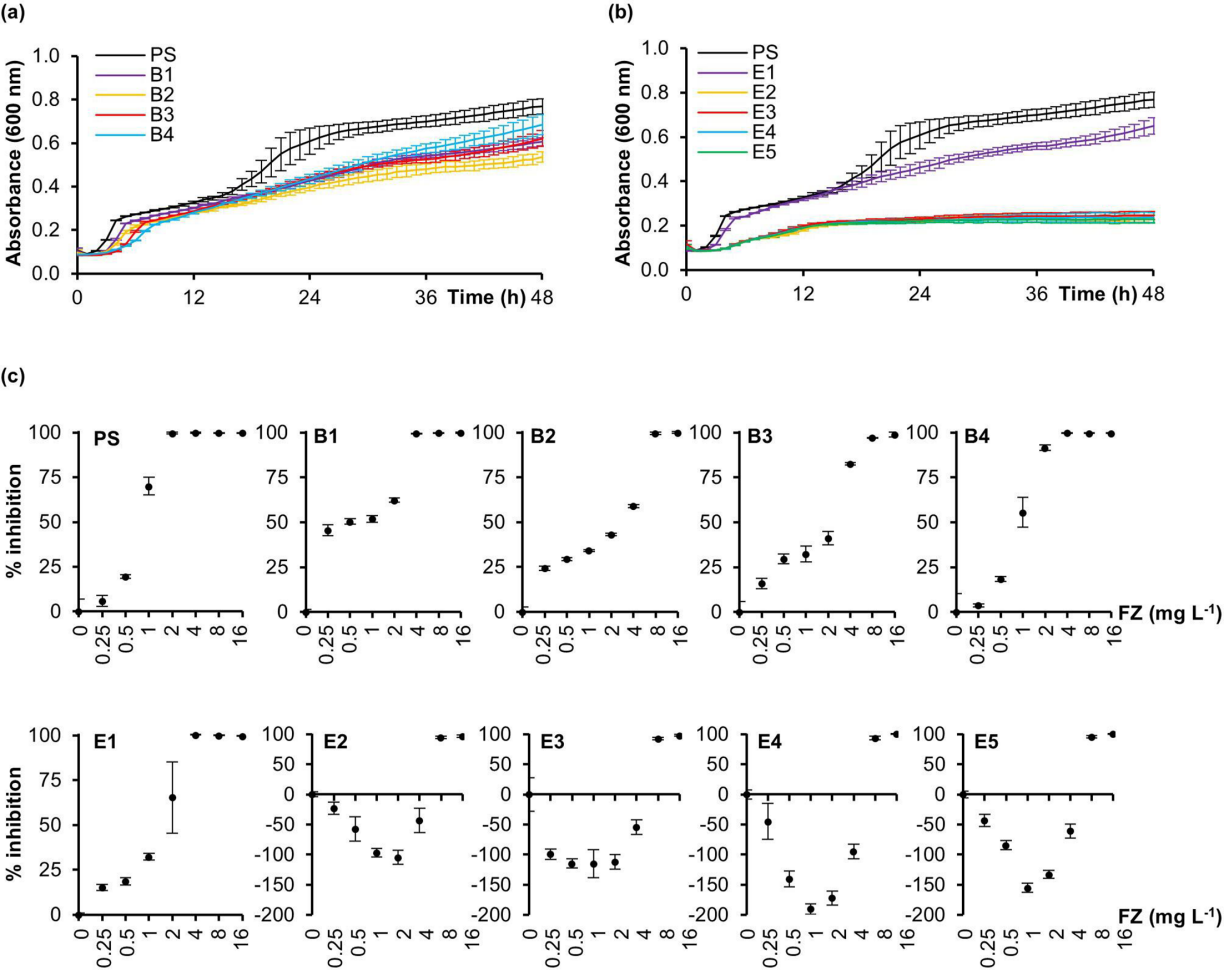
Growth and furazolidone dose-response inhibition profiles of the *ribB*/*ribE* mutants. Growth curves for the (**a**) *ribB* and (**b**) *ribE* mutants and PS were determined by measuring the OD600 every hour for 48 h. (**c**) Furazolidone (FZ) dose-response growth inhibition curves for the *ribB* and *ribE* mutants were determined by a broth microdilution assay at the 18 h time point. Growth inhibition was expressed as a percentage value of the antibiotic-containing culture OD relative to the cultures grown without antibiotics. Data shown are the mean±sd of three replicates.

We next examined the *ribB*/*ribE* mutants’ growth in liquid broth. Most had noticeably slower growth than the PS (Fig. 3a, b). This was particularly severe in the *ribE* TKAG deletion mutants (E2, E3, E4 and E5), which reached stationary phase earlier and at a much lower OD_600_ (~0.2 vs. ~0.6) than E1, the TKAG duplication mutant.

In addition, furazolidone dose-response growth inhibition curves were performed to monitor the inhibitory effect of furazolidone concentration on growth (Fig. 3c). The PS, all *ribB* and the *ribE* TKAG duplication mutant E1 produced a typical sigmoidal dose-response inhibition curve. In contrast, a parabolic curve was observed for RibE TKAG deletion mutants E2, E3, E4 and E5, reflecting substantially improved growth at low furazolidone concentrations, peaking at 0.125× MIC, with a two- to fourfold increased stationary phase OD_600_ relative to the no-furazolidone control.

### Complementation *in trans* reverses the furazolidone resistance and growth defect of the *ribB/ribE* mutants

**Fig. 4.**
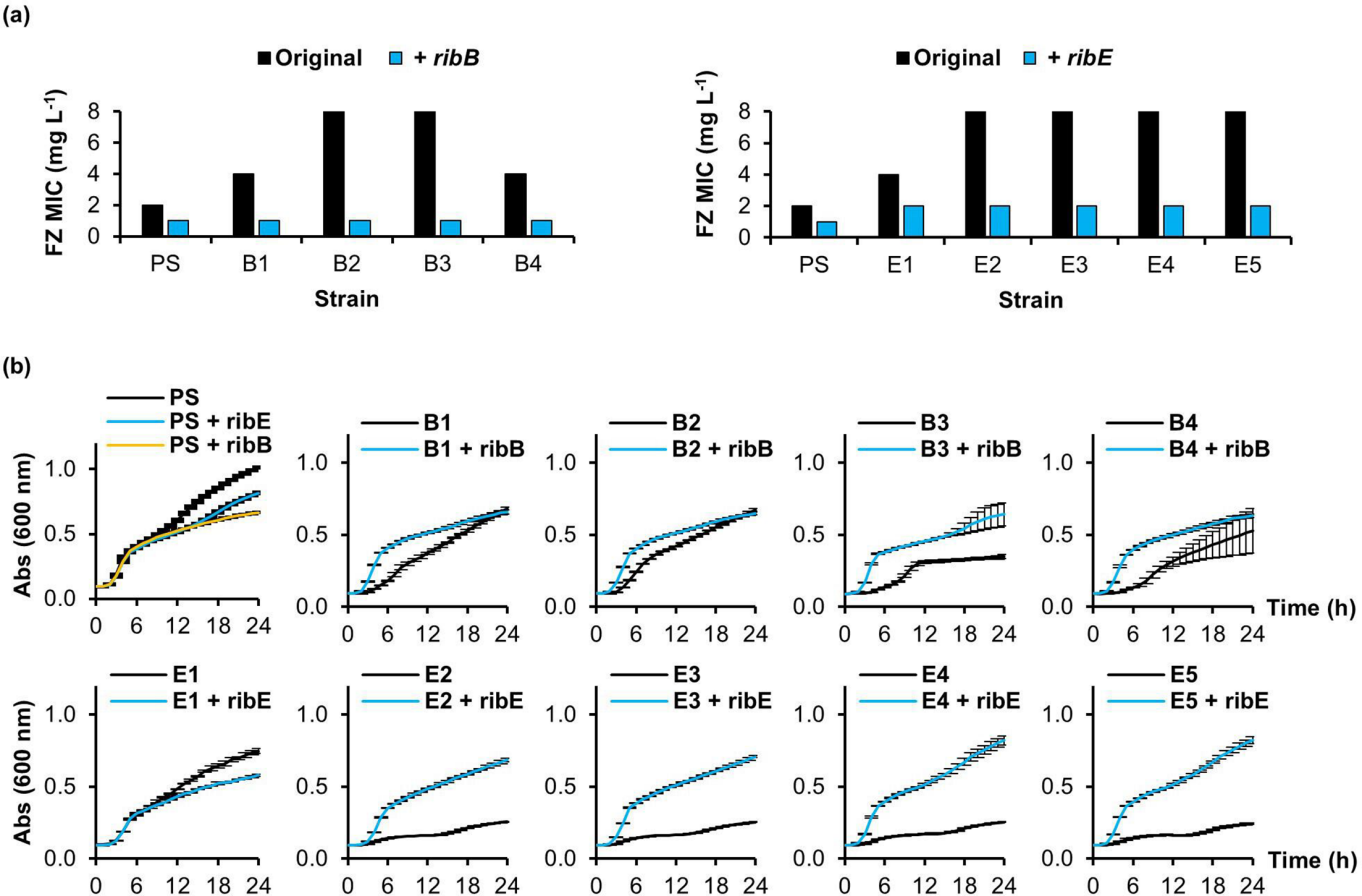
Furazolidone MICs and growth curves for the complemented *ribB/ribE* mutants. (**a**) The change in furazolidone (FZ) MIC upon complementation with either the functional *ribB* (PS, B1, B2, B3, B4) or *ribE* (PS, E1, E2, E3, E4, E5) gene. (**b**) Growth curves of the original and complemented strain at 37 °C. Expression was induced with 0.1 mM IPTG. Absorbance (Abs) at 600 nm was measured every hour for 24 h. Data shown are the mean±sd of three replicates.

We next asked if expressing the corresponding wild-type RibB/RibE proteins from ASKA collection plasmids [9] in the *ribB/ribE* mutants could lower the furazolidone MIC and restore the growth rate relative to the PS. Upon induction with 0.1 mM IPTG, the MIC_FZ_ was reduced to 1 mg l^−1^ for the *ribB* mutants and 2 mg l^−1^ for the *ribE* mutants, which is lower than, or equal to, the PS MIC_FZ_, respectively (Fig. 4a). Notably, the MIC_FZ_ was decreased by twofold if the *ribB* or *ribE* gene was episomally expressed in the PS (Fig. 4a).

Complementation with either *ribB* or *ribE* also improved the growth of all strains except the PS and E1, which had a much less severe growth impairment compared to the other mutants (Fig. 4b). Overall, complementation experiments confirm the causal role of the *ribB*/*ribE* mutations, rather than any secondary mutations identified (Table 2), for the furazolidone resistance and slow growth.

### The *ribB/ribE* mutations cause furazolidone resistance by decreasing the cellular furazolidone-activating nitroreductase activity

RibB and RibE are two essential enzymes in the biosynthesis pathway of riboflavin, the precursor for the cofactors (FMN, FAD) [30,31] of the nitrofuran-activating nitroreductases (NfsA, NfsB, AhpF). To determine whether the *ribB*/*ribE* gene mutations affect the downstream nitroreductase activity, enzymatic assays were conducted on the cell extracts of the PS and some representative isolated mutants: E1 (RibE TKAG duplication), E4 (RibE TKAG deletion, no secondary mutations) and B2 and B3 (IS*1*/*5* insertion within the *ribB* promoter), as well as their corresponding complemented strains.

**Fig. 5.**
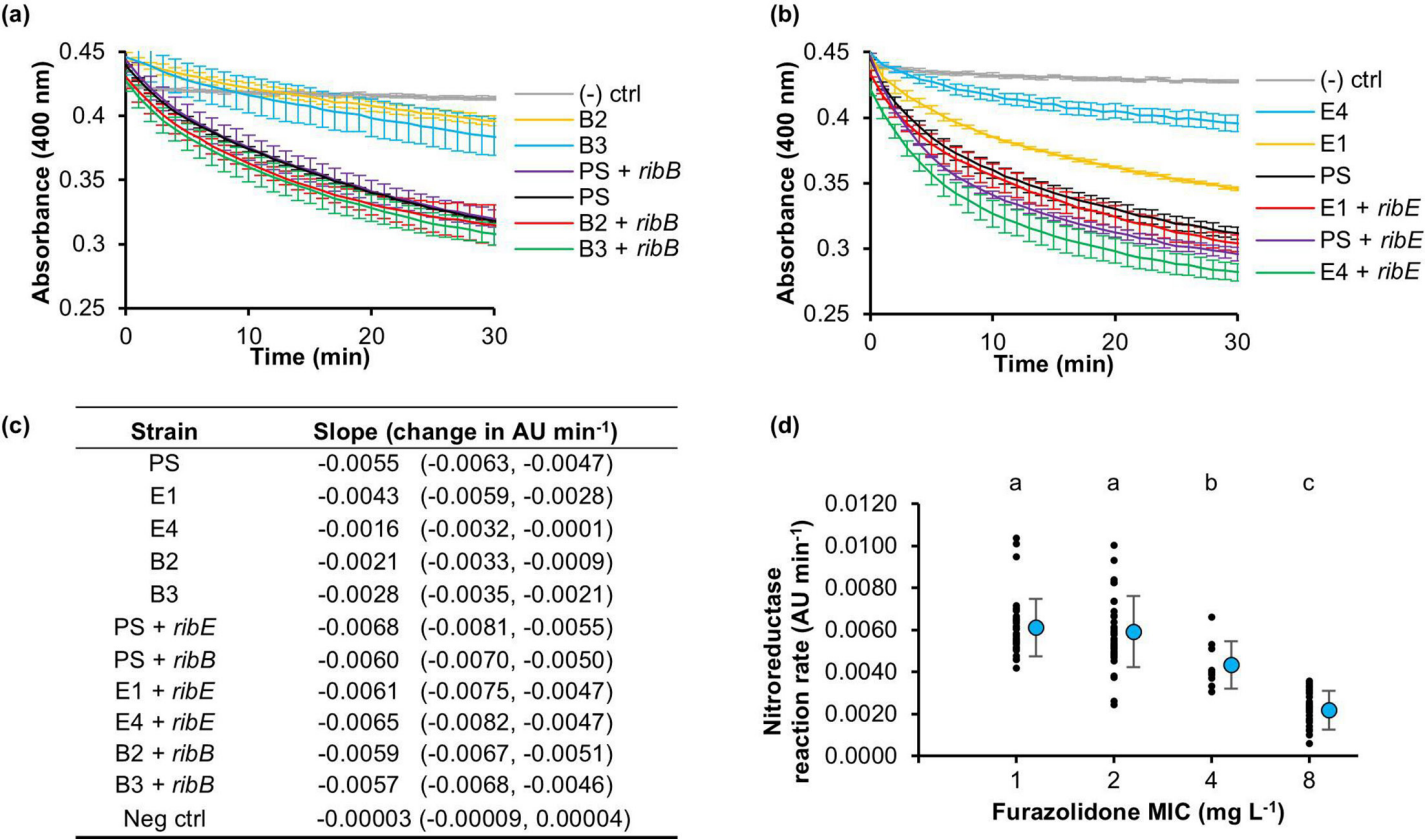
Nitroreductase assays for the *ribB*/*ribE* mutants and their complemented strains. (**a**) Representative graphs showing the reaction progress curve for the nitroreductase assays for the *ribB* mutants and their corresponding complemented strains and (**b**) for the *ribE* mutants and their corresponding complemented strains. Each data point along the curve is the mean ±sd of three replicates. Each reaction contained furazolidone, NADPH and the cellular lysate of the corresponding strain. The absorbance at 400 nm, indicating furazolidone concentration, was measured every minute for 12 h. (–) ctrl: negative control using the buffer in place of the cellular lysate. (**c**) The initial reaction velocity was calculated from three reaction replicates over the first 10 min. The slope and 95% confidence interval are shown. (**d**) The correlation between the initial reaction velocity of the nitroreductase assays and the MIC_FZ_ of the furazolidone-resistant mutants and the corresponding complemented strains. The mean and sd for each MIC value are shown alongside each set of data points. Statistical difference between MIC groups was tested by one-way ANOVA, followed by a post hoc Tukey–Kramer test. Different lowercase letters indicate a significant difference between any two MIC groups (*P*<0.05); AU, arbitrary unit.

The cell lysate nitroreductase activities of the tested furazolidone-resistant mutants were lower than that of the PS (Fig. 5a–c), indicating a lower furazolidone-activating rate. This enzymatic activity was increased to the PS equivalent when the mutants were complemented with the corresponding gene (*ribB* for B2/B3 or *ribE* for E1/E4). Noteworthily, this nitroreductase activity increase correlated with a MIC_FZ_ decrease in these complemented strains (Fig. 5d). Taken together, the *ribB* and *ribE* mutations decreased cellular nitroreductase activity, which subsequently increased furazolidone resistance.

### Effect of *nfsA*/*nfsB* knockout on furazolidone resistance in the *ribB/ribE* mutants

To determine whether the nitroreductase activity decrease was through the major nitroreductases NfsA and NfsB, Δ*nfsA* Δ*nfsB* double-knockout strains were constructed in the *ribB*/*ribE* mutants and the PS by sequential P1-mediated transduction and the MIC_FZ_ determined.

**Fig. 6.**
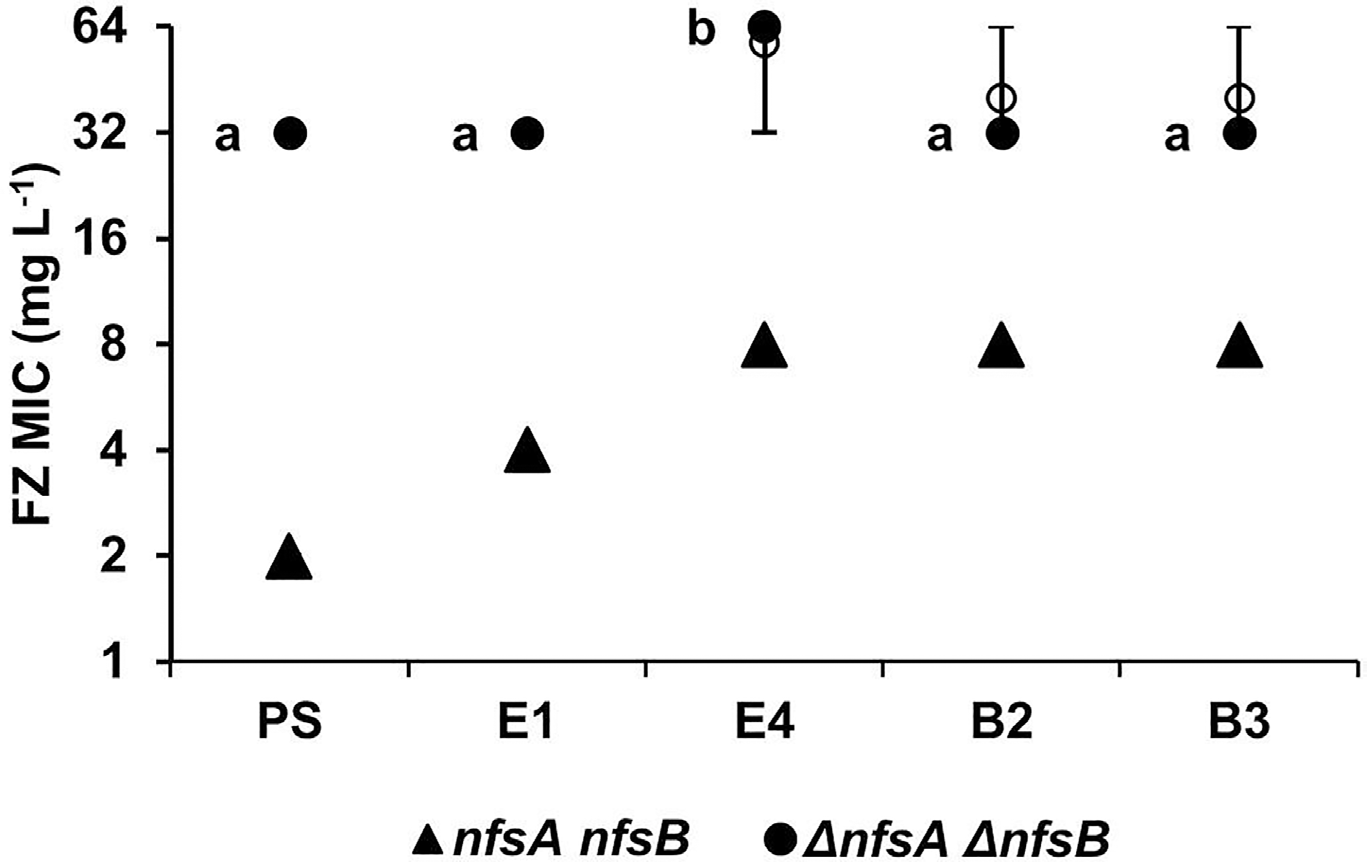
Effect of *nfsA*/*nfsB* knockout on the furazolidone MIC in the *ribB/ribE* mutants. Furazolidone MICs were obtained using standard broth microdilution assays. The strains tested were PS, the E1, E4, B2 and B3 mutants containing wild-type *nfsA* and *nfsB* (solid triangles) and the Δ*nfsA* Δ*nfsB* knockout mutations (solid circles). At least four independent experiments were carried out for each strain. The range, median and mean are shown as bars, filled circles and hollow circles, respectively. Statistical difference between MICs in the Δ*nfsA* Δ*nfsB* knockout mutation strains was tested by the Kruskal–Wallis test, followed by a post hoc Dunn’s test. Different lowercase letters indicate a significant difference between any two MIC groups (*P*<0.05).

In the Δ*nfsA* Δ*nfsB* genetic background, the *ribB*/*ribE* strains were more than twofold closer in furazolidone MIC to the PS than in the wild-type *nfsA nfsB* background (Fig. 6), indicating that the loss of *nfsA* and *nfsB* made the effect of the *ribB*/*ribE* mutations on furazolidone resistance redundant to some extent. Nonetheless, E4 still had increased furazolidone resistance in the Δ*nfsA* Δ*nfsB* genetic background. These findings suggest that the furazolidone resistance mediated by the *ribB*/*ribE* mutations was caused, though not entirely, by decreased NfsA/NfsB nitroreductase activity and that other factors may be involved in the furazolidone resistance.

### Riboflavin supplementation enhances *ribB/ribE* mutant growth but does not affect the furazolidone sensitivity

**Fig. 7.**
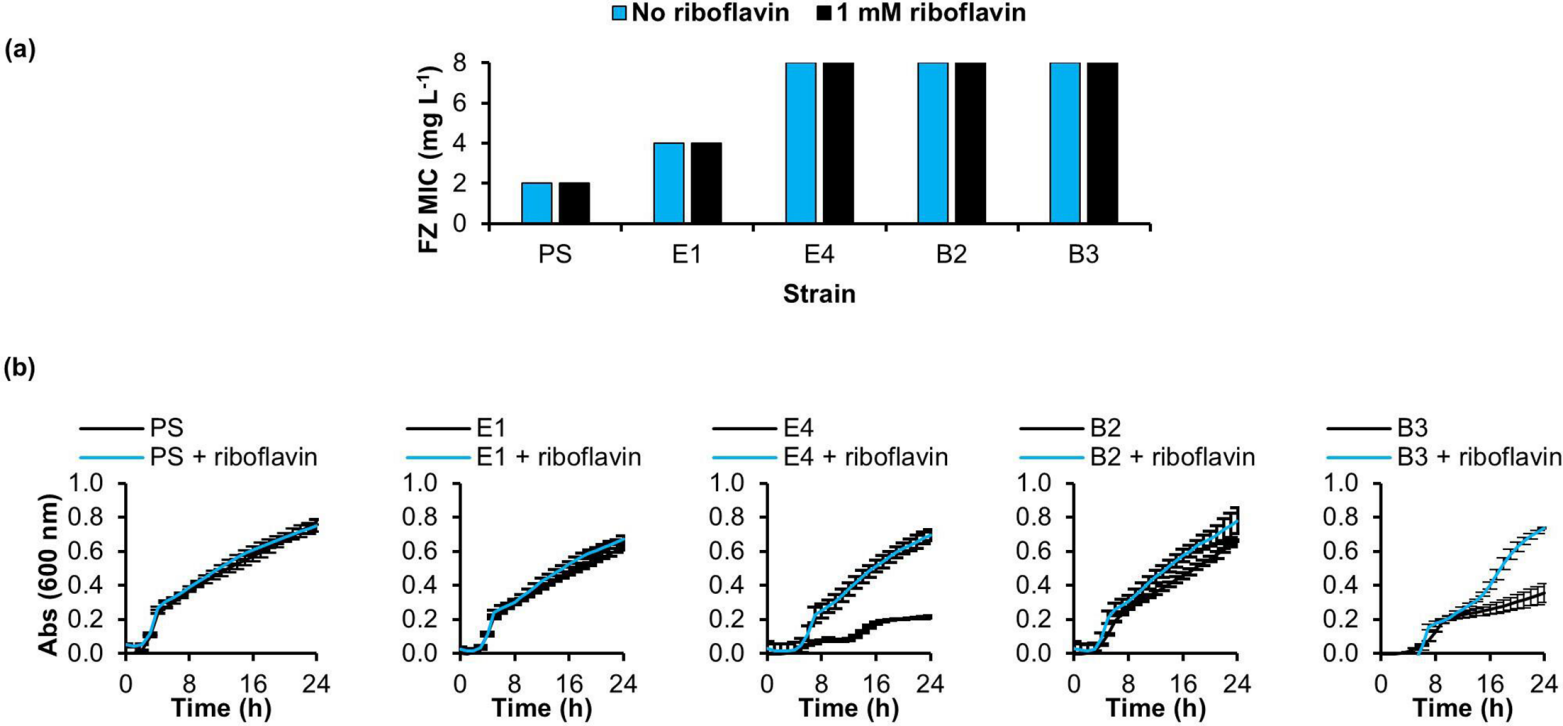
Effect of riboflavin supplementation on furazolidone sensitivity and growth. (**a**) Furazolidone (FZ) MICs and (**b**) growth curves of the furazolidone-resistant mutants and the parent strain upon riboflavin supplementation. Riboflavin was added at a concentration of 1 mM from the preparation of the overnight cultures. The absorbance at 600 nm was measured every hour for 24 h. Data shown are the mean±sd for three replicates.

Given that the *ribB*/*ribE* mutations decrease nitroreductase activity, probably via decreased efficiency in riboflavin biosynthesis (Fig. S2), the precursor of the nitroreductase cofactors (FMN/FAD), we hypothesized that exogenous addition of riboflavin could reverse the furazolidone resistance phenotype in the *ribB*/*ribE* mutants. The effect of 1 mM riboflavin supplementation was therefore investigated in the PS and E1, E4, B2 and B3 strains. We found that while growth was restored to that of the PS, with all strains reaching an OD_600_ of ~0.7 at 24 h (Fig. 7b), all furazolidone MICs remained unchanged (Fig. 7a). This rules out slow bacterial growth as a possible cause to the furazolidone resistance in the *ribB*/*ribE* mutants. Also, it shows that riboflavin supplementation is not viable as a strategy to re-sensitize the *ribB*/*ribE* mutants to furazolidone.

### The TKAG deletion/duplication variants of RibE were found in *E. coli* multidrug-resistant clinical isolates

We next asked if the *ribB*/*ribE* mutations in this study could be found in *E. coli* clinical isolates. Searching the RibE TKAG deletion and duplication variants against the NCBI genome database using blastp [20] retrieved two and three clinical isolates for each mutant, respectively, some of which carry multiple antibiotic resistance genes, such as the strain BLSE9 from France and the strain E2010063_2015 from Australia (Table 3). By contrast, no clinical isolates were found to carry the *ribB* 5′-UTR nucleotide substitution or the promoter region IS*1/5* insertion mutations.

**Table 3.** *E. coli* clinical isolates containing the RibE TKAG deletion or duplication mutations

RibE mutation	Strain	Source	Country	Protein accession no.	Genome accession no.	Antibiotic resistance genes
**TKAG deletion**	ECOL-18-VL-LA-ND-0023	Dog	USA	EFN7364909.1	AATPBR000000000.1	*bla*_CMY-2_
	BLSE9	Human	France	HCK1260674.1	DAIVNX000000000.1	*dfrA12*, *aadA*2, *qacEΔ1*, *sul1*, *bla*_CTX-M-15_, *bla*_OXA-1_, *tetA*, *aac(3)-IIe*, *aac(6’)-Ib-cr6*, *gyrA* (D87N, S83L), *parC* (S80I)
**TKAG duplication**	E2010063_2015	Human	Australia	HBD3720297.1	DAEBRV000000000.1	*catI*, *bla*_TEM-1_, *tetA*, *aph(6)-Id*, *sul2*, *aph(3’)-Ia*
	150832–18	Human	Switzerland	HDK1092331	DANFRG000000000.1	*dfrA5, bla*_TEM-1_, *tetA*, *sul2*, *aph(6)-Id*
	UPEC_003	Human	Poland	OAO67703.1	JSVP00000000.1	*sul2*, *bla*_TEM-1_, *aph(3’’)-Ib*

## Discussion

### Resistance to the furazolidone–vancomycin combination

We have previously shown furazolidone–vancomycin synergy against Gram-negative bacteria [5] and studied the bacterial response to this combination using transcriptomics (RNA-seq) [32]. In this work, we sought to further understand the synergy and potential resistance mechanisms to this combination by selecting and characterizing *E. coli* mutants isolated on furazolidone–vancomycin plates. This screen resulted in mutants with decreased synergy, divided into two groups: increased resistance to furazolidone through *ribB* and *ribE* mutations, or increased resistance to vancomycin (Fig. 1).

Mutations in the *ftsH* gene were the most frequent amongst the increased vancomycin resistance group. Three different mutations of *ftsH* were isolated, all causing a loss of furazolidone–vancomycin synergy and having a collateral sensitivity phenotype (increased vancomycin resistance with increased furazolidone sensitivity) (Table 2, Fig. 1). FtsH is an essential inner-membrane-anchored AAA^+^ protease that degrades specific proteinaceous targets for the removal of misfolded proteins or regulated proteolysis in response to stresses [33]. At least 23 FtsH substrates have been reported, including membrane-anchored and cytoplasmic targets, such as SecY, PspC, KdtA, LpxC, RpoH, SoxS, FolA and Cfa, to name a few [33,35]. It is very likely that the observed phenotypes are due to one or more of these FtsH substrates, whose identity remains to be determined. Future work is warranted to understand the role of the FtsH protein in the furazolidone–vancomycin synergy and collateral sensitivity to furazolidone.

### Mutations in the riboflavin biosynthesis pathway confer resistance to furazolidone

The largest proportion of mutants (9 of 17) had mutations in the essential *ribB* or *ribE* genes, which encode the RibB and RibE proteins in the riboflavin (vitamin B_2_) biosynthesis pathway (Fig. S2). Riboflavin is a precursor to FMN and FAD, cofactors required for the furazolidone-prodrug-activating nitroreductase enzymes NfsA, NfsB and AhpF, in which the former two have a dominant role in drug activation. Using the nitroreductase assay, we established the correlation between the *ribB* and *ribE* mutations, the nitroreductase activity of the cellular lysate and the furazolidone resistance (Fig. 5). The nitroreductase activity affected by the *ribB* and *ribE* mutations could predominantly be attributed to the two major nitroreductases: NfsA and NfsB. The deletion of *nfsA* and *nfsB* from the genomes of isolated *ribB* and *ribE* mutants and their analyses, however, still resulted in increased resistance in the E4 Δ*nfsA* Δ*nfsB* strain in comparison with the Δ*nfsA* Δ*nfsB* parent double mutant, pointing to additional furazolidone-activating enzymes, such as AhpF [24] or undiscovered ones, being involved (Fig. 6).

It is worth mentioning the nature of the *ribB* and *ribE* mutations in this study. Since RibB and RibE are essential enzymes for *E. coli* survival, these mutations may decrease, but not totally abolish, the protein function. The *ribB* mutations were all upstream of the coding sequence, with mutants B2 and B3 having IS*1* and IS*5* insertions, respectively, in the promoter region and mutants B1 and B4 having point mutations in the 5′-UTR of the *ribB* mRNA (Fig. 2). While it is reasonable to assume that disruptions to the promoter region would result in reduced transcription efficiency, how the mutations in the 5′-UTR lead to reduced RibB expression is less clear. The 5′-UTR of the *ribB* mRNA has been previously shown to form an FMN-binding riboswitch or aptamer [30] (Fig. 2b). The binding of FMN to the aptamer prevents the formation of an anti-terminator/anti-sequester stem-loop, allowing the formation of a downstream terminator/ribosome binding site sequester stem-loop, inhibiting expression of *ribB* at both the transcriptional and translational levels [30]. Since the *ribB* mutations in the 5′-UTR found in the B1 and B4 isolates are associated with decreased RibB expression, supported by the increased resistance to furazolidone and restored sensitivity upon *ribB* complementation, these mutations must stabilize, not destabilize, the FMN-bound aptamer to further suppress the RibB translation.

RibE is an essential lumazine synthase in *E. coli* and is a hollow icosahedral complex composed of 60 subunits, assembled from 12 pentamers [36]. All *ribE* mutations isolated here involved the same 12 nucleotides, encoding TKAG (codons 131–134). Mutant E1 had a TKAG duplication, while mutants E2, E3, E4 and E5 had a TKAG deletion. These four residues are located in the interface between two adjacent monomers, involved in substrate binding (Fig. 2c, d) [37], explaining why the enzymatic activity of the corresponding RibE mutant would be negatively impacted.

Notably, the same RibE TKAG deletion has been previously described, in an independent study, where it was selected by, and granted resistance to, nitrofurantoin, another nitrofuran antibiotic [38]. This and the fact that all the *ribE* mutants were independently isolated from separate plates in our screen indicate that the *ribE* mutation to gain nitrofuran resistance is highly constrained and predictable.

In agreement with the essentiality of *ribB* and *ribE*, all mutants have shown slower growth than the parent, with the *ribE* TKAG deletion mutants being the most affected. When riboflavin (metabolite downstream from the RibB and RibE catalysed reactions in the biosynthesis pathway) was supplemented in the medium, the growth defect was rectified. Most interestingly, however, riboflavin did not abolish furazolidone resistance, showing that slow bacterial growth has no role in the furazolidone resistance of the *ribB*/*ribE* mutants and ruling out the possibility of riboflavin supplementation to re-sensitize the *ribB*/*ribE* mutants to furazolidone. This observation likely reflects the complex functional and regulatory roles of riboflavin. For example, riboflavin could be preferentially used by essential enzymes supporting bacterial growth, but not for functional restoration of the NfsA and NfsB enzymes. Another curiosity observed in this work is the growth-stimulatory effect of furazolidone at sublethal concentrations on the slow-growing *ribE* TKAG deletion mutants. This observation is in favour of the direct activity of furazolidone as an electron donor or acceptor in essential biological processes that are normally dependent on FMN/FAD.

### Co-presence of furazolidone-resistant *ribE* mutations and other AMR genes in *E. coli* clinical isolates

The RibE TKAG^131–134^ deletion has been previously identified in *E. coli* [38]; hence, the *ribE* gene has been added as a target when surveying nitrofurantoin resistance in clinical and environmental isolates [39,41]. We have searched the TKAG^131–134^ RibE deletion variant identified here against the NCBI database and found two clinical isolates, from the USA and France, where the latter also contained several other antibiotic resistance determinants (Table 3). Similarly, we found three *E. coli* clinical isolates containing the TKAG^131–134^ duplication with the co-occurrence of other AMR determinants (Table 3).

The detection of these *ribE* mutations in clinical isolates, despite these mutations having significant fitness costs on the host, is concerning. This study provides evidence for three possible causes: (i) the fitness cost can be compensated by external nutrients, such as riboflavin supplementation that improves the growth of the *ribE* mutants without re-sensitizing the cell to furazolidone (Fig. 7). As excess riboflavin is excreted via the kidneys into urine, and nitrofurans are commonly used to treat urinary tract infections, riboflavin would most likely be available at the infection site for urinary tract infections [42], (ii) the *ribE* mutant may be co-selected with other antimicrobial resistance factors upon exposure to other antibiotics (Table 3) and (iii) the *ribE* mutant ‘feeds’ on furazolidone at sub-inhibitory concentrations via an unknown mechanism (Fig. 3c). An alternative scenario is that compensatory mutations occur to improve the cell fitness through bypassing the decreased riboflavin biosynthesis pathway. Future work looking into this aspect of the *ribE* mutants is important to help devise a strategy to counter-select against the nitrofuran-resistant *ribE* mutants in the clinical setting.

In conclusion, we have shown that mutations decreasing transcription and/or translation of *ribB* and *ribE* genes in the riboflavin biosynthesis pathway can confer resistance to the furazolidone–vancomycin combination through decreasing nitroreductase activity. Mutations in the *ribE* gene arise in the clinical setting despite a significant fitness cost to the host, likely due to their ability to regain wild-type growth levels in the presence of riboflavin, whilst retaining a furazolidone-resistant phenotype and/or being co-selected with other antimicrobial resistance genes. Given that nitrofurans have been commonly used for urinary tract infection therapy in recent years, *ribE* mutants are expected to become a formidable obstacle in the treatment of infections caused by multi-resistant Gram-negative pathogens.

## Supplementary material

10.1099/mgen.0.001356 Uncited Supplementary Material 1.

## Abbreviations

AMR: antimicrobial resistance
CAMH: cation-adjusted Mueller Hinton
FAD: flavin adenine dinucleotide
FICI: fractional inhibitory concentration index
FLP: flippase
FMN: flavin mononucleotide
NADPH: nicotinamide adenine dinucleotide phosphate
NCBI: National Center for Biotechnology Information
PS: parental strain
Tris-HCl: tris(hydroxymethyl)aminomethane hydrochloride
